# Neuromodulation of the Cardiac Autonomic Nervous System for Arrhythmia Treatment

**DOI:** 10.3390/biomedicines13071776

**Published:** 2025-07-21

**Authors:** Benjamin Wong, Yuki Kuwabara, Siamak Salavatian

**Affiliations:** 1 Department of Anesthesiology and Perioperative Medicine, University of Pittsburgh, Pittsburgh, PA 15261, USA; 2 Department of Bioengineering, University of Pittsburgh, Pittsburgh, PA 15219, USA; 3 Department of Medicine, Division of Cardiology, University of Pittsburgh, Pittsburgh, PA 15213, USA

**Keywords:** neuromodulation, atrial arrhythmias, ventricular arrhythmias

## Abstract

This review explores current and emerging neuromodulation techniques targeting the cardiac autonomic nervous system for the treatment and prevention of atrial and ventricular arrhythmias. Arrhythmias remain a significant cause of morbidity and mortality, with the autonomic nervous system playing a crucial role in arrhythmogenesis. Interventions span surgical, pharmacological, and bioelectronic methods. We discuss the range of neuromodulation methods targeting the stellate ganglion, the spinal region, the parasympathetic system, and other promising methods. These include stellate ganglion block, stellate ganglion ablation, cardiac sympathetic denervation, subcutaneous electrical stimulation, thoracic epidural anesthesia, spinal cord stimulation, dorsal root ganglion stimulation, vagus nerve stimulation, baroreflex activation therapy, carotid body ablation, renal denervation, ganglionated plexi ablation, acupuncture, and transcutaneous magnetic stimulation. Both preclinical and clinical studies are presented as evidence for arrhythmia management.

## 1. Introduction

Cardiac arrhythmias, both atrial and ventricular, affect millions and remain a leading cause of morbidity and mortality worldwide. Atrial fibrillation (AF) accounts for more than half of all arrhythmia cases, currently affecting over 2.2 million individuals in the United States, a number projected to rise to approximately 5.6 million by 2050 [1]. AF significantly increases the risk of stroke, imposing a substantial burden on the healthcare system [2]. On the other hand, ventricular arrhythmias (VAs) are the leading cause of sudden cardiac death, responsible for an estimated 180,000 to 250,000 deaths annually in the U.S. [3]. Although current therapies, including antiarrhythmic drugs, rate control agents, and anticoagulants, are widely used, they often have limited efficacy, notable side effects, and fail to provide durable outcomes. Interventional approaches such as catheter ablation and implantable cardioverter-defibrillators (ICDs) offer more effective alternatives but are associated with procedural risks and are not curative, as their long-term effectiveness may diminish over time [4,5].

**Figure 1:**
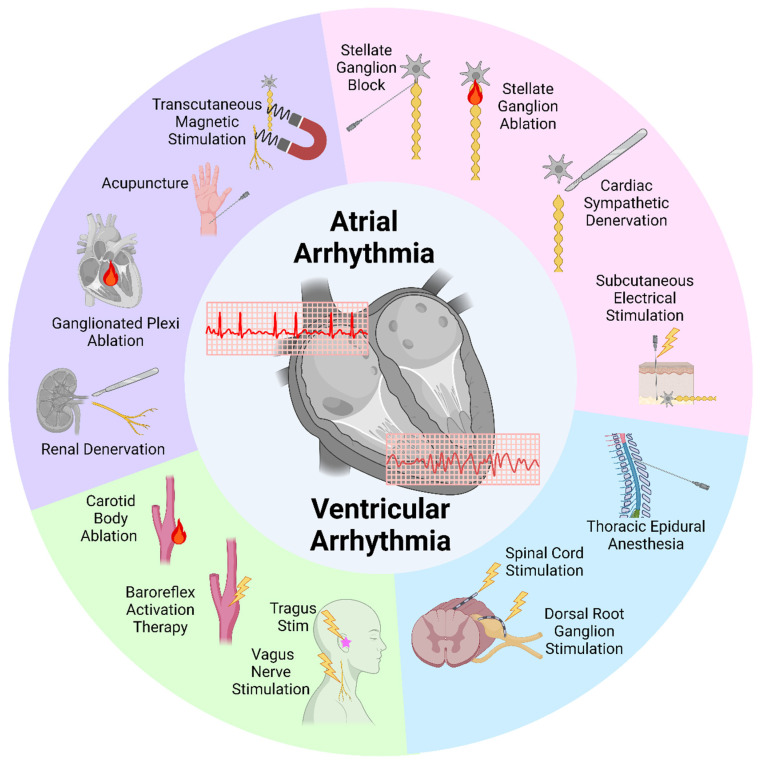
Neuromodulation therapies to treat atrial and ventricular arrhythmias. Therapies range from pharmacological methods to surgical procedures to specialized devices targeting the stellate ganglion (pink), the spinal cord region (blue), the parasympathetic nervous system (green), and other (purple). Created in BioRender. Wong, B. (2025) https://BioRender.com/5kqb43n.

The cardiac autonomic nervous system (CANS) consists of efferent sympathetic and parasympathetic pathways as well as afferent pathways that regulate heart function. These neural pathways are interlinked in a cardio-neural hierarchy [6,7,8]. Within this hierarchical structure, afferent and efferent signals from the sympathetic and parasympathetic nervous systems modulate the heart and vasculature during every cardiac cycle [9,10]. In cardiac disease, including arrhythmias, the activation of afferent/sensory pathways results in sympathoexcitatory and parasympathetic inhibitory reflexes, which are mediated through the CANS hierarchy [11,12,13,14]. Several preclinical and clinical studies have shown that sympathetic excitation and/or vagal withdrawal play a crucial role in the pathophysiology of cardiac disease, especially arrhythmia [14,15,16,17]. As the imbalance of the CANS is one of the mechanisms in the development and triggering of arrhythmia, modulating the CANS can be a therapeutic option [9,18,19,20]. Recent meta-analyses have shown the impact of surgical neuromodulation methods used in prior studies to treat cardiac arrhythmias [21]. This review overviews recent preclinical studies and clinical trials in which neuromodulation has been performed to treat atrial or ventricular arrhythmias (Figure 1).

## 2. Targeting the Stellate Ganglion

### 2.1. Stellate Ganglion Block

The stellate ganglia (SG) are neural networks located in the lower part of the neck on the sympathetic chain on both sides of the body [22]. The spinal preganglionic sympathetic neurons synapse with postganglionic neurons in the SG, delivering efferent sympathetic output to the heart muscle [23]. This offers an ideal therapeutic site since the spinal sensory afferents implicated in arrhythmogenesis travel through the SG. The SG block (SGB) can be carried out by infusing anesthetic agents, so the surrounding fibers and possibly neurons can be inhibited and help attenuate both afferent and efferent neurotransmission at the SG. Recent reviews discussed the variety of antiarrhythmic drugs and SGB interventions utilized for electrical storm and VAs [24,25,26,27].

For anesthetic agents, bupivacaine, lidocaine, and ropivacaine are mostly used, but injections of toxins, such as botulinum toxin A (BoNT-A), have also been explored for SGB [24,28,29]. Lidocaine exhibits a higher binding affinity for inactivated fast sodium channels; however, its use is limited by time- and dose-dependent neurological side effects [24]. In patients experiencing ventricular arrhythmia storm, SGB using either single-injection or continuous-infusion techniques significantly reduced arrhythmia burden. However, the continuous-infusion approach achieved a greater reduction in arrhythmia episodes, maintained a similar safety profile, and eliminated the need for repeat interventions [30]. While lidocaine is fast-acting, bupivacaine and ropivacaine are long-acting agents [24]. These pharmacokinetic differences allow for strategic combinations to achieve tailored anesthetic effects depending on the desired onset and duration of nerve blockade. BoNT-A and conventional anesthetics differ in onset, duration, and safety. Traditional agents like lidocaine offer rapid but short-term relief (minutes to hours), while BoNT-A has a delayed onset but can last weeks to months [31,32]. Local anesthetics may cause systemic toxicity if misapplied, whereas BoNT-A risks include localized muscle weakness and unintended spread [24,28]. Clinical evidence for BoNT-A in pain or autonomic modulation remains limited. Future SGB studies should directly compare BoNT-A and conventional anesthetics in controlled trials and explore their mechanisms and optimal applications.

Imaging guidance with ultrasound [30,33,34,35] was introduced as a novel alternative compared to conventional SGB with anatomic landmarks [36]. The standard approach targets the left SG; however, the right SG may be considered in cases of recurrent arrhythmias [26]. Nevertheless, it has been suggested that right SG blockade does not yield additional benefit and may increase the risk of bilateral phrenic nerve paralysis [24].

Early studies on canines showed an increase in ventricular fibrillation (VF) thresholds after SGB [23]. This was then translated to human patients and confirmed [37]. A later rat study showed left SGB lowering arrhythmia scores and even reducing the infarct size [38]. Mechanistically, a study on rabbits posits that left SGB increases the electrophysiological stability of the ventricular myocardium by prolonging 90% of the monophasic action potential duration of the epicardium, decreasing the dispersion of repolarization, and increasing the effective refractory period along with ventricular threshold [39]. They demonstrated that SGB prolonged the atrial effective refractory periods on both the right and left sides of the atria. SGB extensively targets the treatment of VAs. Many clinical case reports have shown the effectiveness of ceasing or reducing episodes of VAs [15,34,40,41,42]. Meng et al. reviewed the efficacy of the SGB in managing VAs and reported that SGB showed a significant decrease in VA burden and the number of external and implanted defibrillator shocks [43]. After the SGB procedure, 80.6% of patients in the study survived discharge [43]. Another study compared the efficacy of SGB between single and continuous injections in patients with VA storm [30]. The number of sustained VAs within 24 h was significantly reduced with the continuous infusion compared to the one before treatment. More recently, Baldi et al. evaluated the efficacy of preemptive percutaneous SGB for managing electrical storm [44]. They compared one group that received SGB prior to antiarrhythmic drug administration (early SGB) with a group that received it afterward (delayed SGB) [44]. Their findings indicated that early SGB significantly reduced the number of arrhythmic episodes treated within the first hour, decreased the need for defibrillation, and potentially mitigated the overall incidence of electrical storm [44].

In a recent multicenter study, Savastano et al. (STAR study) reported improved management of VAs in 131 patients following percutaneous SGB [26]. Notably, 106 patients (92%) experienced a reduction in the number of treated arrhythmic episodes [26]. The study did not identify differences between image-guided and anatomical landmark techniques for SGB [26]. Similarly, another multicenter study by Chouairi et al. demonstrated that SGB was associated with a decreased burden of VT/VF and a reduced need for defibrillation [27]. The number of VT/VF episodes significantly declined (*p* < 0.001) within 24 h following SGB [27]. A key limitation of this study was the absence of a control group, highlighting the need for future investigations to evaluate potential placebo effects [27].

Overall, the effect of SGB on atrial arrhythmias (AAs) has not been well-studied, even less so in humans [45]. Leftheriotis et al. investigated the effect of unilateral SGB on the electrophysiology of the atrium and AF induction in paroxysmal AF patients [46]. They found that SGB decreased both AF inducibility and duration [46]. A recent case study by Groenendyk et al. described a 73-year-old woman with rapid AF who received SGB via ropivacaine infusion [47]. After 13 days of hospitalization, her left ventricular ejection fraction improved from approximately 10% to 25% [47]. This represents the first published in-human case reporting the use of SGB for the management of acute AF [47].

### 2.2. Stellate Ganglion Ablation

As discussed, SG is one of the neuromodulation targets for arrhythmia. The justification of SG ablation is the same as SGB. It can be performed using continuous radiofrequency under fluoroscopic guidance, instead of injecting anesthetic drugs as the SGB [48]. Animal studies show SG ablation prevents VAs induced by myocardial infarction (MI) [49,50]. There are also studies on AAs in dogs showing the potential of SG cryoablation [51]. This involves inserting a cooled catheter into the ablation target to freeze and destroy it [51]. Bilateral SG cryoablation decreased the sympathetic tone and SG nerve activity associated with the onset of AAs [51]. A later study confirmed this and showed that subcutaneous nerve activity can help estimate cardiac sympathetic tone and the effects of cryoablation [51].

There are not many clinical studies targeting SG for ablation. The study by Rao et al. showed that radiofrequency SG ablation treated patients with electrical storm [48]. Bilateral SG ablation was shown to be effective in treating electrical storm, with survival at discharge for all patients at a mean follow-up of 22 ± 8 months [48].

The potential risks and complications associated with this approach have not been well reported to date. They are presumed to be similar to those of SGB, particularly concerning acute procedural risks and hemodynamic effects. However, long-term outcomes remain uncertain and warrant further research, perhaps by performing prospective trials.

### 2.3. Cardiac Sympathetic Denervation

It has been widely known that the autonomic nervous system plays a crucial role in the genesis and maintenance of VAs. Cardiac-related preganglionic somas reside within the intermediolateral cell column in the spinal cords; these somas project axons to other postganglionic somas at the stellate, middle cervical, mediastinal, and intrinsic cardiac ganglia [52]. Additionally, these ganglia consist of neural networks with afferent neurons and local neurons in the circuit. These intrathoracic ganglia function within a hierarchy for cardiac control, where peripheral elements are mainly involved in cardio–cardiac reflexes [8]. There is both experimental and clinical justification that fatal arrhythmias can be mitigated by cardiac sympathetic denervation (CSD), especially in the case of augmented sympathetic activity [53,54,55]. The safety and feasibility of video-assisted thoracoscopic surgical CSD have been reported, which can provide a minimally invasive approach with minimal perioperative complications [56].

In rats, CSD was shown to reduce the susceptibility of AF by altering the expression of connexin 43, known as a major gap junction protein found in the heart [42]. In patients with a history of AAs, CSD was shown not to change the AAs’ burden [57]. While adjunctive therapies are promising, along with CSD [45], more studies need to be conducted to understand the direct impact of CSD on AF in human patients. In terms of VF, many past studies explore the effects of CSD in different models: it has been shown to increase the inducibility threshold of VF, treat QT syndrome, decrease the number of cardiac events, and decrease the number of shocks from preoperative implantable defibrillators [58,59,60]. A recent review paper summarized similar outcomes, showing a significant reduction in corrected QT (QTc) interval after left CSD (LCSD) with no evidence of arrhythmias and reduced beta-blocker dependence [61]. A study published in 2021 showed a decreased mean QTc interval of 14 long QT syndrome patients from 506.2 ms to 476 ms after CSD, and they concluded bilateral CSD (BCSD) to be an effective and safe treatment [62]. Another report by Bourke et al. showed that among nine patients with LCSD, three patients had complete success with LCSD [63]. A recent review and meta-analysis by Hanna et al. detailed the minimized rate of ICD shocks and occurrence of VAs [64]. Furthermore, Assis et al. reported the effectiveness of BCSD to the burden of ventricular tachyarrhythmias [65]. Freedom from sustained ventricular tachycardia (VT) or ICD shock was 60% and 54.5% after BCSD at 1 and 4 years. A recent study by Ahmed et al. further investigated BCSD as a treatment for VA in patients with prior unsuccessful premature ventricular contraction (PVC) ablation procedures [66]. The study demonstrated that BCSD was associated with significant reductions in PVC burden, improvements in left ventricular ejection fraction, and the discontinuation of antiarrhythmic medications [66].

To compare the effectiveness between LCSD and BCSD after one year, Vaseghi et al. reported the shock-free survival rates from sustained VT/ICD shock among 121 patients who underwent left or bilateral CSD: 58.2% and 50.4%, respectively [67]. CSD reduced the burden of ICD shocks between study entry and follow-up.

Although CSD has demonstrated substantial effectiveness in reducing arrhythmia burden, patient responses remain highly variable. Contributing factors to this heterogeneity include underlying cardiac pathology (e.g., ischemic versus nonischemic cardiomyopathy, channelopathies), severity of heart failure (with advanced New York Heart Association [NYHA] class predicting poorer outcomes), and procedural variations (such as left versus bilateral CSD and the inclusion of Kuntz nerve ablation) (Table 1).

### 2.4. Subcutaneous Nerve Stimulation

Subcutaneous nerve activity and superficial skin sympathetic nerve activity recorded in the chest wall correlate with SG activity [75,76,77]. This technique is likely originated from acupuncture. Stimulation at the Xinshu acupoint (BL15), located approximately 5 cm lateral to the spine at T5 level, has been shown to alter mRNA expression in sympathetic ganglia in a rat model [78]. Electrodes are positioned within the thoracic subcutaneous tissue near this region to deliver stimulation [78,79]. Preclinical work demonstrated the feasibility and efficacy of this novel technique to show a reduced sympathetic tone (3.5 mA, 10 Hz, 14 s ON and 1.1 min OFF) [78] and reduced fibrosis in persistent AF in a canine model (3.5 mA, 10 Hz, 20 s ON and 60 s OFF) [79]. Clinically, a prospective randomized trial to test whether chronic subcutaneous nerve stimulation can decrease AF burden was recently completed (STALL-AF [Using Electrical Nerve Stimulation to Control Atrial Fibrillation]; NCT04529941. The results of this study are not available yet and are expected to be released soon. In terms of VA, dogs with acute MI and increased risk of VT underwent subcutaneous nerve stimulation of the SG [80]. The outcomes showed decreased mean SG nerve activity after MI [80]. However, further clinical studies need to be performed to establish optimal stimulation protocols and long-term safety in humans.

## 3. Targeting the Spinal Cord and Dorsal Root Ganglion

### 3.1. Thoracic Epidural Anesthesia

Thoracic epidural anesthesia (TEA) is a common practice for relieving perioperative pain but has begun developing as a therapeutic method for arrhythmia treatment. The sympathetic pathway from the spinal cord travels through the sympathetic chain, then to the heart; thus, the spinal cord is a prime target for interrupting sympathetic signals that may cause arrhythmia [81]. TEA has been shown to provide partial or selective sympathetic blockade by impeding spinal segments targeting the ventricles [16]. Therefore, TEA could inhibit fibers proximally to both the right and left SG, thereby reducing efferent output to the heart. TEA has been shown to increase the VF threshold during acute myocardial ischemia while also prolonging ventricular repolarization and the effective refractory period [82,83]. Additionally, TEA has been shown to suppress the effects of sympathoexcitation on the shortening of the ventricular activation recovery interval and the spatial heterogeneity of repolarization in the heart [84]. Applying local anesthetic epidurally causes almost immediate selective sympatholysis by reducing adrenergic output. The dose of TEA can be adjusted according to the patient’s response [85]. The efficacy of this approach is influenced by factors such as dose concentration and catheter placement [86]. For instance, a study evaluating three different concentrations of ropivacaine TEA found that the highest concentration produced the most significant reduction in systemic vascular resistance [87]. Precise catheter insertion is critical, as incorrect placement has led to major complications in patients, including hematoma formation, nerve injury, local anesthetic systemic toxicity, ineffective analgesia, or unintended high/block spread [86].

In preclinical studies, TEA reduced the myocardial excitability induced by SG stimulation in pigs. It also showed TEA stabilizing the electrical wave, such as activation recovery interval, and markers of arrhythmogenesis, such as dispersion of repolarization [84]. The anti-arrhythmic effects of TEA are also attributed to increases in ventricular effective refractory periods, decreases in the slope of restitution, and decreases in the border zone heterogeneity [88]. One of the first clinical applications was reported in 2005, showing the effectiveness of TEA in reducing episodes of electrical storm [89]. Another case report appeared to show similar outcomes with TEA [90]. Both cases highlighted the usefulness of TEA to manage ventricular storms with little hemodynamic changes, even under other pharmacological agents being used. Do et al. reported a case series with patients who underwent TEA, showing a reduction of ≥80% in arrhythmia burden [85]. Another study showed that patients who had TEA followed by LSCD all survived to hospital discharge [63].

### 3.2. Spinal Cord Stimulation

Spinal cord stimulation (SCS) affects the cardiac nervous system centrally and peripherally [6,91]. SCS can intercept cardiac afferent signals from the heart and suppress efferent output from the intermediolateral region of the spinal cord within the spinal cord. This sympathetic efferent suppression leads to reduced cardiac sympathoexcitation back to the heart through the stellate ganglion, thus resulting in cardioprotective effects such as improvements in cardiac function and reduction in arrhythmias [14,92,93]. GABA-mediated pathways have been examined for SCS to work within the spinal cords in the pain field [94], but they still need further research in the cardiac field. SCS leads are typically situated in the epidural space percutaneously, and a programmable pulse generator (IPG) is implanted in the pocket in the back for delivering the stimulation. In terms of practical stimulation methods, no optimal stimulation method has been confirmed yet. SCS has shown effective pain relief in many clinical studies by applying traditional low-frequency stimulation at 50 Hz [95]; however, recently, kilohertz high-frequency stimulation has shown better pain relief in line with patients’ satisfaction with less perception and less paresthesia [96,97]. For the purpose of arrhythmia treatment, there is even less known about the optimization of SCS. For example, how long the stimulation should be delivered is another question, and no optimal period has been determined yet. High-frequency stimulation may represent a preferable approach, as it is associated with reduced paresthesia [98,99]. Kilohertz-frequency SCS has also been shown to produce longer-lasting inhibitory effects on myocardial ischemia-related spinal neurons compared to conventional low-frequency SCS [100]. Furthermore, computational modeling [101,102], combined with preclinical studies to validate these findings, could facilitate the identification of optimal stimulation parameters prior to clinical application.

Animal studies with SCS are showing promising effects in mitigating VAs and excitatory arrhythmogenesis. In pigs, low-frequency 50 Hz SCS targeting the T3/T4 spinal level was shown to reduce VTs and measures of cardiac electrophysiology and arrhythmogenesis, such as activation recovery interval and repolarization time shortening, dispersion of repolarization, and arrhythmia score [103]. The same research group posits that SCS reduces VAs by mitigating glial cell activation and activating GABA signaling pathways [104,105]. Another mechanism explored is the regulation of norepinephrine release; SCS significantly reduced norepinephrine levels associated with arrhythmogenesis in a canine cardiac ischemia model [106]. There is extensive preclinical research from physiological and mechanistic aspects to prevent VAs by SCS [105,107]; however, there are few clinical studies, possibly because of the invasive nature of the procedure and the use of anticoagulants in these cardiac patients. In the SCS HEART clinical study, SCS was shown to improve heart failure symptoms and to protect against VAs [92]. A study in 2015 showed that SCS minimized episodes of VT and fibrillation in a patient with severe electrical storm [108]. There are also several clinical studies focusing on the relief of angina pectoris with SCS [109,110].

In an AF canine model, SCS lengthens the atrial effective refractory period and decreases AF burden and inducibility [111]. When inducing atrial tachyarrhythmias and bradycardias in dogs, SCS obtunds the excitatory signals triggering arrhythmogenesis [112]. These effects are speculated to be modulated through decreased activity in the ICNS [113]. Autonomic remodeling of the right atrial GP and left SG caused by fast atrial pacing-induced AF was shown to be attenuated by SCS in the canine model [93]. In a recent clinical study, temporary SCS demonstrated efficacy for suppressing post-operative AF after coronary artery bypass grafting [114].

### 3.3. Dorsal Root Ganglion Stimulation

Cardio-spinal neural reflexes pass through the dorsal root ganglion (DRG) while traveling from primary afferent neurons to the dorsal horn at the thoracic level of the spinal cord [115]. As such, the DRG offers an accessible target for bioelectronic neuromodulation [116]. DRG stimulation (DRGS) electrodes can be placed in the prone position using fluoroscopic guidance, with the electrode positioned over the DRG in the dorsal region of the foramen [117]. There is evidence of DRGS relieving pain in preclinical studies [118,119,120], but there are few studies related to arrhythmia treatment. Currently, there are only animal studies for the application of DRGS in reducing arrhythmogenesis. In recent pig studies, thoracic DRGS decreased sympathoexcitation and ventricular arrhythmogenesis [121]. Also, it has been shown that thoracic DRGS reduced ventricular arrhythmogenesis using both low (20 Hz) and high (1 kHz) frequencies [122].

## 4. Targeting Parasympathetic Systems

### 4.1. Vagus Nerve Stimulation

The vagus nerve is the 10th cranial nerve, which contains both parasympathetic efferent and afferent axons in the nerve [123]. Based on these anatomical features, vagus nerve stimulation (VNS) can modulate the cardiac autonomic nervous system [6]. The VNS lead needs to be placed surgically in the neck, and the IPG is implanted in the chest for direct VNS [124]. Through this direct way of stimulation, both afferent and efferent axonal paths are regulated [125,126]. Additionally, a noninvasive transcutaneous VNS approach at the tragus has been explored and has shown effectiveness in modulating cardiac autonomic imbalances [127,128,129]. There are no optimal stimulation parameters confirmed yet, but Ardell et al. mentioned a potential configuration to ensure the most effective delivery of invasive stimulation [91].

Many animal studies explore the effects of VNS on AF [130]. In one study, low-level VNS was shown to increase the threshold when trying to induce fibrillation with stimulation of other parts of the CANS, such as the GP, atrium, and pulmonary veins [131]. In canines, it is shown that VNS targets specific neuronal populations of the intrinsic cardiac system to attenuate the potential for AF [132].

There is controversy on whether VNS has antiarrhythmic effects or proarrhythmic effects. An early study had indicated that strong VNS (above 100% sensory thresholds) would induce AF, so moderate intensities (less than 80% of thresholds) can deliver therapeutic effects without risk of arrhythmia [133,134]. Additionally, distinct neurotransmitters have been shown to mediate different outcomes. The pro-arrhythmogenic effects of high-intensity VNS are driven by elevated levels of acetylcholine and vasoactive intestinal peptide, whereas the anti-arrhythmogenic effects observed at lower stimulation intensities are mediated by nitric oxide or phosphatidylinositol-3-kinase [134]. Clinical VNS applications normally use moderate intensities that cause mild-to-undetectable slowing of the heart rate [135] and can modulate heart rate variability depending on target location and stimulation parameters [136]. A more recent report by Stavrakis et al. showed that low-level VNS at a frequency of 20 Hz and 50% below the threshold for 72 h reduced the episodes of post-operative AF in patients undergoing cardiac surgery compared to the control group [137]. The same group also showed that transcutaneous VNS at a frequency of 20 Hz and 50% below the threshold for one hour decreased pacing-induced AF [138].

In terms of VAs, preclinical data support that VNS is effective in VA prevention [139,140]. Invasive VNS in canine models has shown a suppression of VA [139,141]. A recent study by Hu et al. has shown that MI rats treated with implantable remote-controlled VNS had significantly reduced incidence and duration of VAs after four weeks [142]. Additionally, noninvasive VNS of the tragus has also been shown to reduce arrhythmia inducibility in canines [143].

### 4.2. Baroreflex Activation Therapy

Baroreceptors are mechano-sensitive neurons in both the aortic arch and carotid sinus that modulate blood pressure and the autonomic nervous system [144]. Stimulating baroreceptors can stimulate afferent parasympathetic nerves, leading to an increase in parasympathetic tone and a decrease in sympathetic tone [145]. Stimulation of the carotid sinus is the common method for baroreceptor stimulation.

There is only preclinical evidence for baroreflex activation therapy to be effective in AAs [133]. In porcine models, carotid baroreceptor stimulation at levels commonly used for hypertension showed an increase in vagal tone, resulting in atrial effective refractory period shortening and increased AF inducibility [146]. On the contrary, in the canine model, reducing the magnitude of stimulation below the threshold to lower blood pressure decreased right atrial Ganglionated plexi (GP) neural activity and increased the atrial effective refractory period; thus, AF inducibility was reduced [147,148].

Similarly to AAs, there is a lack of clinical evidence for treating VAs, although a preclinical study showed a reduction in episodes of premature ventricular contractions during acute myocardial ischemia and VF [149].

### 4.3. Carotid Body Resection or Ablation

The carotid bodies (CBs) are bilaterally positioned at the bifurcation of the carotid arteries, strategically placed to ensure sufficient oxygen supply to the brain [150]. They are sensitive to small changes in blood oxygen, flow, carbon dioxide, and pH [151,152]. The CBs have strong reflex effects on the respiratory and cardiovascular systems [150,153]. This afferent system is normally inactive at resting conditions, but when hypoxia occurs, the CBs are triggered, increasing ventilation and sympathetic activity, while contributing to periodic breathing during sleep [154,155].

As it relates to arrhythmias, there are very few studies on CB ablation. However, in patients with heart failure, the overactivation of sympathetic tones is associated with chemoreflexes in the CBs [156]. Thus, the removal of these bodies can mitigate sympathetic activation. In a preclinical study, CB ablation was shown to reduce the number of AAs in rats with chronic hypoxia mimicking obstructive sleep apnea [157]. A study found that early CB ablation treatment in a rat model of chronic heart failure reduced ventricular arrhythmogenesis by eliminating the carotid chemoreflex drive associated with the heart failure state [158]. However, no clinical studies are exploring the therapeutic effects of CB ablation on cardiac arrhythmias.

## 5. Other Potential Neuromodulation Techniques

### 5.1. Renal Denervation

Renal sympathetic nerves contribute to cardiac function and sympathetic outflow to the kidneys [159]. Efferent sympathetic outflow triggers the release of renin, the increase in tubular sodium reabsorption, and the decrease in renal blood flow [160]. Many clinical trials have been performed to demonstrate the effectiveness of renal denervation to treat persistent hypertension by likely modulating sympathoexcitation in hypertension [17,161,162]. However, due to sympathetic nerves present in renal arteries and the role of sympathetic nerves in triggering arrhythmia, there is potential for renal denervation (RDN) to hinder arrhythmogenesis [163].

RDN led to a reduction in postinfarction VAs in pigs [164]. In canines, another study found similar results, concluding that RDN contributed to a reduction in neural remodeling in the heart and SG associated with MI, and thus, consequent arrhythmias [165]. VT can be a target for RDN, with one of the first clinical applications reported in 2012 [166]. Later, it was shown that adjunctive RDN was more effective than only VT ablation in the prevention of VT/VF without any procedure-related complications [167]. A more recent study by Bradfield et al. demonstrated the efficacy of radiofrequency-based RDN in treating refractory VT. Patients who underwent RDN as an adjunctive treatment to CSD had a reduction in ICD therapies when comparing 6 months pre-RDN versus 6 months post-RDN, with ICD shocks being a significant decrease [168].

Additionally, it has been shown that RDN can be effective in the treatment of AF [169]. RDN reduced the burden of AF at 12 months after the treatment [170]. Catheter ablation through the isolation of the pulmonary vein is an option for patients who have displeasing responses to pharmacological treatment for AF. Although more effective than drug treatment for minimizing AF recurrence [171], ablation has a 20% to 50% failure rate, resulting in repeat procedures [172,173]. Steinberg et al. investigated whether RDN, when combined with pulmonary vein isolation (PVI), could enhance long-term antiarrhythmic efficacy [174]. Among 283 patients, freedom from AF, tachycardia, or flutter after 12 months was observed in 56.5% of patients undergoing pulmonary vein isolation alone and in 72.1% of those undergoing PVI plus RDN [174].

### 5.2. Ganglionated Plexus Ablation

GP are autonomic ganglia on the epicardium, primarily within the epicardial fat [175]. Studies have shown that the GP consists of a heterogeneous neuronal population. This includes afferent, interconnecting, and efferent neurons, where interconnecting neurons account for most of the neural components in the GP [9]. Additionally, the GP acts as the communication center between the intrinsic and the extrinsic CANS, responsible for coordinating signal trafficking between afferent and efferent circuits [8]. In the body, the four major atrial GPs reside close to the pulmonary veins (PVs), and each innervates a respective PV and the nearby atrial myocardium [176,177]. The interconnections of the GP converge to the sinus node as well as the atrioventricular (AV) node via the anterior right GP and inferior right GP, respectively [178]. In humans, these neural pathways similarly connect the GP with the PVs, sinus node, and AV node [179].

GP ablation is performed either at their estimated anatomical locations or the location identified using high-frequency stimulation [180]. The best technique to perform GP ablation remains undecided.

In animal studies, there tend to be conflicting results on the arrhythmic effects of GP ablation. GP ablation was shown to be potentially proarrhythmic in canines [181]. Studies have shown that GP ablation increases the risk of VAs after myocardial ischemia [182], and GP ablation alone is not superior in maintaining AF-free survival [183]. A recent study demonstrated promising results, showing that targeted anatomical and high-frequency stimulation-guided epicardial pulsed field GP ablation prolongs the atrial effective refractory period and reduces both AF inducibility and duration [184].

Long-term effects of GP ablation have not been explored heavily, but a study posits that introducing other therapies along with GP ablation could help reduce the risks of arrhythmogenesis [185]. They showed that GP ablation and ligament of Marshall ablation mitigated paroxysmal arrhythmias [185]. Randomized clinical studies of the additional influence of GP ablation during PV isolation have been conflicting [186,187]. In a randomized study involving 242 patients undergoing PV isolation, Katritsis et al. found that those assigned to additional anatomically based GP ablation experienced a 47% relative reduction in the risk of recurrent atrial tachycardia or AF over a two-year follow-up period [188]. On the other hand, in the AF Ablation and Autonomic Modulation via Thoracoscopic Surgery (AFACT) study, GP ablation was performed thoracoscopically as an adjunct to PV isolation. Anatomical identification of GPs was guided by high-frequency stimulation to evoke vagal responses [187]. However, the study found that selective GP ablation without concomitant PVI did not significantly improve outcomes in patients with persistent AF [187]. These findings suggest that GP ablation alone may be insufficient as a standalone therapy for persistent AF but may have a synergistic effect when combined with PV isolation, potentially enhancing the efficacy of surgical ablation strategies.

Several factors may account for the conflicting outcomes observed with GP ablation, including variations in ablation techniques, differences in the AF subtypes targeted, and diverse underlying patient conditions [189,190]. Emerging technologies, such as advanced mapping systems, may help enhance the effectiveness of GP ablation [191,192].

### 5.3. Acupuncture

Acupuncture has a long history in the East, especially in China [193]. Acupuncture has been performed to treat cardiovascular diseases, spanning from hypertension to angina, and it is gaining popularity in the West [194]. There are numerous clinical studies suggesting the protective effect of acupuncture for cardiac ischemia and remodeling [195,196,197,198,199]. To perform acupuncture, needles are inserted into specific acupoints, each with its own specific targets and therapeutic effects [200]. In general, the effects of acupuncture appear more pronounced when the patient has a sympathetic tone greater than normal at baseline [201]. Additionally, electroacupuncture has been explored by inducing electrical stimulation at the needle’s target [202,203,204]. This methodology involves sending tolerable electrical stimulation through the needles at a desired frequency and intensity [204]. When it comes to cardiac arrhythmias, there are even fewer but promising studies for the application of acupuncture [205,206,207].

In rodents, multiple studies have shown that acupuncture reduces VAs. One study showed that targeting the PC6 pressure point with electroacupuncture reduced the arrhythmia score in rats during myocardial ischemia–reperfusion [206]. Zuo et al. similarly found that acupuncture attenuated the sympathetic tone by reducing the expression of inhibitors responsible for VAs [207]. Clinically, acupuncture has decreased the rate of AF recurrence in persistent AF patients [208]. The recurrence of AF can also be quantified as the AF burden, which accounts for the frequency of AF and how often the patient is in a state of AF [200]. Compared to control AF patients with a 54% recurrence rate, patients receiving acupuncture and amiodarone treatment had significantly lower recurrence rates of 35% and 27%, respectively [209]. Thus, acupuncture is approaching a similar therapeutic of medications.

### 5.4. Transcutaneous Magnetic Stimulation

Transcutaneous magnetic stimulation (TcMS) is a technique that takes advantage of the plasticity of a patient’s nervous system and can contribute to excitation or inhibition depending on stimulation parameters [210,211]. This methodology is important not only due to its ability to change neural circuitry but also because it is noninvasive and nondestructive. TcMS is commonly used for treating pain and depression [212]. Also, it has been shown to impact heart rate variability and cardiac rhythm, causing minor parasympathetic activation [213]. However, TcMS can be targeted not only within the parasympathetic nervous system, such as the tragus, but also within the sympathetic nervous system, such as the SG [214].

TcMS of parasympathetic trunks was shown to suppress AF [214,215]. Preclinical evidence supports the promise of this non-invasive treatment to suppress AF, showing that in the canine model, the electromagnetic field stimulation reduced the burden of the AF caused by rapid atrial pacing [216]. Clinically, patients with paroxysmal AF were shown to have a reduced chance of AF and shorter episodes of pacing-induced AF using low-level electromagnetic fields [217]. In terms of the sympathetic nervous system, in a randomized trial of patients with VT storms who were treated with TcMS of the left SG, the burden of VAs decreased without adverse events [210,214]. Also, a more recent study showed a similar outcome with transcutaneous magnetic stimulation, with a reduction in episodes of VAs at 24 h post-treatment [20].

## 6. Conclusions

The neuromodulation of the CANS holds significant promise for the treatment of both atrial and ventricular arrhythmias. Among the available approaches, SGB and CSD currently have the most robust clinical evidence, particularly in the management of VAs. However, their invasive nature, variability in long-term efficacy, and the lack of large-scale randomized controlled trials present significant barriers to widespread adoption. Device-based therapies, such as VNS and SCS, are also promising; however, they face challenges including invasiveness, the need to establish optimal stimulation protocols, uncertainty regarding long-term efficacy, and a lack of large-scale randomized controlled trials. Future studies should prioritize the development of minimally invasive alternatives with comparable efficacy, while also addressing the critical need for rigorous randomized clinical trials to evaluate long-term outcomes, durability of response, and patient selection strategies across different neuromodulatory approaches.

## Figures and Tables

**Table 1:** Key clinical studies on cardiac sympathetic denervation for refractory ventricular arrhythmias and heart failure.

Study (Year)	Population (n)	Age (Years)	Cardiac Pathology	CSD Approach	Primary Outcomes
Hofferberth et al. [68] (2014)	24	5 weeks to 27 years	Long QT syndrome, catecholaminergic polymorphic VT, idiopathic VT	Left	73% arrhythmia reduction; 55% arrhythmia-free
Vaseghi et al. [67] (2017)	121	55 ± 13	Structural heart disease, refractory VT	Left or bilateral	1-year freedom from VT/ICD shock: 58%; ICD shocks reduced
Cai et al. [69] (2019)	19	60.3 ± 14.6	Structural heart disease, recurrent VA	Left (14), bilateral (5)	VA/ICD therapies reduced; 3-year heart transplant/death-free: 52.6%
Shah et al. [70] (2019)	173	54.6 ± 2	Structural heart disease, refractory VA	82% bilateral	Event-free: 58–100%; 28% complication rate (mostly minor)
Barwad et al. [71] (2021)	65	50 ± 18	Structural heart disease, refractory VT	Surgical (mostly bilateral)	92% reduction in defibrillation shocks; 2-year ICD shock/death-free: 51.5%
Ertugrul et al. [62] (2021)	14	8–19 years	Long QT syndrome, catecholaminergic polymorphic VT, other	Bilateral + Kuntz ablation	Arrhythmia reduction; no major complications
Yalin et al. [72] (2021)	10	61.6 ± 19.6	Nonischemic cardiomyopathy, r efractory VA	Left (6), bilateral (4)	VA/ICD shocks reduced; 2 deaths (not CSD-related)
König et al. [73] (2022)	21	63.7 ± 14.4	Structural heart disease, refractory VA	90.5% bilateral	77% ICD shock-free at 9 mo; 9.5% major complications
Brady et al. [74] (2024)	32	62 ± 11.6	Systolic heart failure, refractory VA	Bilateral (27), unilateral (5)	1-year survival: 61.4%

## Data Availability

Not applicable.

## Abbreviations

The following abbreviations are used in this manuscript:

CANS	Cardiac Autonomic Nervous System
SG	Stellate Ganglion
SGB	Stellate Ganglion Block
BoNT-A	Botulinum Toxin A
VA	Ventricular Arrhythmia
VF	Ventricular Fibrillation
AA	Atrial Arrhythmia
AF	Atrial Fibrillation
MI	Myocardial Infarction
CSD	Cardiac Sympathetic Denervation
QTc	Corrected QT
LCSD	Left Cardiac Sympathetic Denervation
BCSD	Bilateral Cardiac Sympathetic Denervation
ICD	Implantable Cardioverter-Defibrillator
VT	Ventricular Tachycardia
TEA	Thoracic Epidural Anesthesia
SCS	Spinal Cord Stimulation
IPG	Implantable Programmable Pulse Generator
DRG	Dorsal Root Ganglion
DRGS	Dorsal Root Ganglion Stimulation
VNS	Vagus Nerve Stimulation
GP	Ganglionated Plexi
CB	Carotid Body
RDN	Renal Denervation
PV	Pulmonary Vein
AV	Atrioventricular
AFACT	AF Ablation and Autonomic Modulation via Thoracoscopic Surgery
TcMS	Transcutaneous Magnetic Stimulation
